# Therapeutic potential of active components of saffron in post-surgical adhesion band formation

**DOI:** 10.1016/j.jtcme.2021.01.002

**Published:** 2021-01-20

**Authors:** Mohammad-Hassan Arjmand, Milad Hashemzehi, Atena Soleimani, Fereshteh Asgharzadeh, Amir Avan, Saeedeh Mehraban, Maryam Fakhraei, Gordon A. Ferns, Mikhail Ryzhikov, Masoumeh Gharib, Roshanak Salari, Sayyed Hadi Sayyed Hoseinian, Mohammad Reza Parizadeh, Majid Khazaei, Seyed Mahdi Hassanian

**Affiliations:** a Medical Plants Research Center, Basic Health Sciences Institute, Shahrekord University of Medical Sciences, Shahrekord, Iran; b Iranshahr University of Medical Sciences, Iranshahr, Iran; c Department of Clinical Biochemistry, Faculty of Medicine, Mashhad University of Medical Sciences, Mashhad, Iran; d Department of Physiology, Faculty of Medicine, Mashhad University of Medical Sciences, Mashhad, Iran; e Metabolic Syndrome Research Center, Mashhad University of Medical Sciences, Mashhad, Iran; f Student Research Committee and Department of Medical Genetics, Faculty of Medicine, Mashhad University of Medical Science, Mashhad, Iran; g Immunology Research Center, Inflammation and Inflammatory Diseases Division, School of Medicine, Mashhad University of Medical Sciences, Mashhad, Iran; h Brighton & Sussex Medical School, Division of Medical Education, Falmer, Brighton, BN1 9PH, UK; i Division of Pulmonary and Critical Care Medicine, Washington University, School of Medicine, Saint Louis, MO, USA; j Department of Pathology, Faculty of Medicine, Mashhad University of Medical Sciences, Mashhad, Iran; k Department of Pharmaceutical Sciences in Persian Medicine, School of Persian and Complementary Medicine, Mashhad University of Medical Sciences, Mashhad, Iran; l Orthopedic Research Center, Mashhad University of Medical Sciences, Mashhad, Iran

**Keywords:** Saffron, Crocin, Crocetin, Post-surgical adhesion band formation, Fibrosis, Inflammation, APC, activated protein C, DSS, dextran sodium sulfate, HE, Hematoxylin & Eosin, IP, intera-peritoneal, MDA, malondialdehyde, PSAB, post-surgical adhesion band, PDGF, platelet-derived growth factor, TGF-β, transforming growth factor-beta, α-SMA, α-smooth muscle actin, SOD, superoxidase dismutase, TAA, thioacetamide

## Abstract

**Background:**

Abdominal adhesions are common and often develop after abdominal surgery. There are currently no useful targeted pharmacotherapies for adhesive disease. Saffron and its active constituents, Crocin and Crocetin, are wildly used in traditional medicine for alleviating the severity of inflammatory or malignant disease.

**Purpose:**

The aim of this study was to investigate the therapeutic potential of the pharmacological active component of saffron in attenuating the formation of post-operative adhesion bands using different administration methods in a murine model.

**Material method:**

saffron extract (100 mg/kg), Crocin (100 mg/kg), and Crocetin (100 mg/kg) were administered intraperitoneally and by gavage in various groups of male Wistar rat post-surgery. Also three groups were first treated intra-peritoneally by saffron extract, Crocin, and Crocetin (100 mg/kg) for 10 days and then had surgery. At the end of the experiments, animals sacrificed for biological assessment.

**Result:**

A hydro-alcoholic extract of saffron and crocin but not crocetin potently reduced the adhesion band frequency in treatment and pre-treatment groups in the mice given intra-peritoneal (i.p) injections. Following the saffron or crocin administration, histological evaluation and quantitative analysis represented less inflammatory cell infiltration and less collagen composition, compared to control group. Moreover, the oxidative stress was significantly reduced in treatment groups.

**Conclusion:**

These findings suggest that a hydro-alcoholic extract of saffron or its active compound, crocin, is a potentially novel therapeutic strategy for the prevention of adhesions formation and might be used as beneficial anti-inflammatory or anti-fibrosis agents in clinical trials.

**Taxonomy:**

Abdominal surgeries/post-surgical adhesions.

## Highlights of the findings and novelties

• This study investigates the protective effects of saffron in preventing post-surgical adhesion band (PSAB) formation.
• Saffron and crocin but not crocetin (i.p) decrease PSAB through anti-inflammatory and fibrinolytic mechanisms.
• Our results support the therapeutic potential of saffron against PSAB-associated complications.

## Graphical abstract

**Figure:**
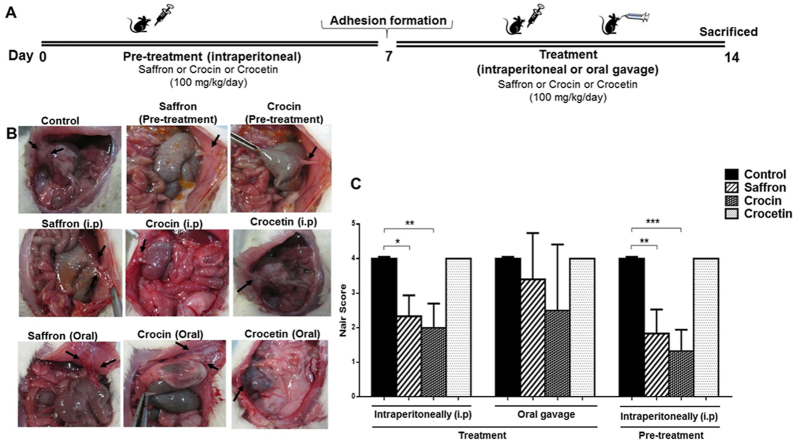

## 1 Introduction

Intraperitoneal adhesion formation is a serious worldwide complication of surgery developing between abdominal wall and intra-abdominal surfaces following peritoneal irritation. This condition is highly prevalent (up to 90%) in patients undergoing open gynecological pelvic or open abdominal surgery.^1^^,^^2^ Intestinal obstruction,^3^ female infertility,^4^ chronic abdominal and pelvic pain^5^ are the most important consequences of post-operative adhesion bands. Placement of some solid barriers such as Seprafilm® is a standard strategy for adhesion bands prevention which needs to accurately recognize the damage sites.^6^ Predicting where these fibrotic bands may develop is a barriers usage limitation encouraging the researchers to find an efficient therapeutic method.^6^ Therefore, identifying the exact mechanisms involved in adhesion band formation would help to progress the treatment process.

It is generally accepted that pathological adhesions generation is the end-result of improper healing process in peritoneal cavity.^7^ In post-operative physiological responses, immune cell-released inflammatory cytokines, as well as platelet-derived growth factor (PDGF), and fibroblast-induced transforming growth factor-beta (TGF-β) are increased in peritoneum.^8^, ^9^, ^10^, ^11^ It has been shown that inflammation and fibrosis play key roles in the pathogenesis of adhesion band formation.^11^, ^12^, ^13^ We previously investigated the effects of anti-fibrotic or anti-inflammatory compounds, EW-7197 ^14^ and activated protein C (APC)^15^ on post-surgical adhesions, representing the hopeful results in reduction of these fibrotic bands. Although there are extensive studies on post-surgical adhesion band formation, our knowledge of pathogenic mechanism of this surgery-associated complication is still limited.

The plant-based therapies to improve pathologic conditions have been used for centuries and counted as potential treatments due to low complications and minimal toxicities. The huge parts of FDA-approved components are contained the therapeutics agents derived from natural components.^16^^,^^17^ The natural products continue to play an important role in drug discovery programs for different diseases. Using different technical approaches,^18^^,^^19^ studies showed the effectiveness of these components for various disorders such as carcinomas,^20^, ^21^, ^22^, ^23^, ^24^, ^25^, ^26^ diabetes,^27^ various inflammatory and fibrotic conditions.^28^, ^29^, ^30^, ^31^, ^32^ One of these natural products was isolated from Crocus sativus, a flowering plant in the Iridaceae family and is commonly known as saffron.^33^

Saffron, the dried stigmas of Crocus sativus, is cultivated in Iran and other countries such as Spain and turkey^34^ and has a wide range of activities including oxytocic, anti-carcinogenic,^35^^,^^36^ and etc. . There are several active pharmacological components in saffron including crocin and crocetin that are responsible for the biological effects of saffron.^37^^,^^38^ Saffron contains essential mineral and various important vitamins. Generally, due to anti-aging and anti-oxidant function of saffron, its daily consumption is very high in many parts of the world. Also, the ability of saffron, crocin or crocetin to reducing adverse effects of chemotherapeutic components showed their drug modulator activities.^39^ Animal studies showed low or non-toxicity of saffron and its extract.^40^ A temporary immunomodulatory activity with no renal, hepatic, hematological, or any side effects were reported in sub-chronic use of 100 mg/day saffron in a randomized controlled trial study.^41^ Studies revealed that these compounds elicit anti-inflammatory and anti-fibrotic responses by decreasing the inflammatory mediators in different animal models^42^, ^43^, ^44^ which is consistent with our previous work, showing anti-inflammatory effects of crocin in mice model.^45^ In line with this, saffron potently decreased the level of Malondialdehyde (MDA) and inflammatory cytokines in bleomycin-induced pulmonary fibrosis.^46^ Similarly, crocin significantly reduced the over-expression of fibrotic molecules such as collagen 1a, α-smooth muscle actin (α-SMA), and TGF-β in hepatic fibrosis condition.^47^

In this study, we investigated the protective effects of saffron, crocin, or crocetin in preventing post-surgical adhesion band formation in animal models. Our results showed that saffron or crocin (i.p) could suppress the formation of adhesion bands through anti-inflammatory, anti-oxidant and anti-fibrosis function in rat model, suggesting the therapeutic potential of this non-toxic compounds against post-surgery-associated complications.

## 2 Material and methods

### 2.1 Animal

Animal experiments were conducted in according to the guideline for Care and Use of Laboratory Animals from Mashhad University of Medical Sciences (MUMS). Animals were purchased from pasture institute (Tehran, Iran) and were subjected to controlled conditions of temperature (20 ± 2 °C) and humidity (50%). The animals were housed in a standard cage in a 12 h light-dark cycle room with free access to both food and water. This study was approved by ethical committee of Mashhad University of Medical Sciences, ethical approval number: 960917.

### 2.2 Reagents

Pure crocin and crocetin were purchased from BuAli Pharmacological Research Center (MUMS, Mashhad, Iran). Hydro-alcoholic extract of Saffron was isolated by Maceration method as described.^48^^,^^49^ Reagents need for hematoxylin and eosin, malonyl dialdehyde (MDA), total thiol, and catalase were purchased from Sigma Co (Saint Louis, MO).

### 2.3 Surgical abrasion model

Surgical method for adhesion band formation was performed as described.^50^^,^^51^ Briefly, the animals were anesthetized by intraperitoneal injection of ketamine.^52^ After shaving and preparation of the site of surgery with 1% antiseptic povidone-iodine solution, the abdominal cavity was opened with 1.5 cm incision. The anterior cecal surface was gently rubbed until partial petechial hemorrhages were generated and the interior surface of abdomen was damaged using a medical electric scalpel. The cecum placed in front of damaged surface of abdomen and the muscular layer was sutured. Intraperitoneal adhesion formation occurs with this procedure probability above 90% between damaged surfaces.^53^ The evaluation of post-surgical adhesion bands was performed according to the previously explained adhesions grade criteria by Nair et al.^54^ in a blinded fashion, assessing the incidence and frequency of fibrotic bands. The detailed of Nair scoring system is shown in Table 1.

**Table 1:** Adhesion score system for macroscopic evaluation (Nair’s et al.).

**0 to 4**	**Adhesion grade**
**0**	Complete absence of adhesion
**1**	Single band of adhesion, between viscera or from viscera to abdominal wall
**2**	Two bands, either or from viscera to abdominal wall
**3**	More than two bands, between viscera or viscera to abdominal wall
**4**	Viscera directly adherent to abdominal wall, irrespective of number and extent of adhesive bands

### 2.4 Experimental design

Rats were randomly divided into the following groups of 6 rats in each group: 1) Control group (animals with surgical abrasion received saline as vehicle intraperitoneally (i.p)); 2) animals with surgical abrasion treated intraperitoneally with 100 mg/kg/day saffron extract; 3) animals with surgical abrasion treated intraperitoneally with 100 mg/kg/day crocin; 4) animals with surgical abrasion treated intraperitoneally with 100 mg/kg/day crocetin.^55^, ^56^, ^57^, ^58^

To investigate whether type of administration, i. p or gavage could affect protective effects of these compounds, we also add the following groups: 5) animals with surgical abrasion treated by oral gavage with 100 mg/kg/day saffron extract; 6) animals with surgical abrasion treated by oral gavage with 100 mg/kg/day crocin; 7) animals with surgical abrasion treated by oral gavage with 100 mg/kg/day crocetin.

Next, we were interested in evaluating the preventive potential of these compounds in post-surgical adhesion band formation. In this case, before the surgery, we consider more groups: 8) animals were first treated intra-peritoneally with 100 mg/kg/day saffron extract for 10 days and then had surgery; 9) animals were first treated intra-peritoneally with 100 mg/kg/day crocin for 10 days and then had surgery; 10) animals were first treated intra-peritoneally with 100 mg/kg/day crocetin for 10 days and then had surgery.

All animals weighted before surgical abrasion and during the experiment. At the end of the experiments, animals sacrificed for biological assessment. The excised tissue samples were quickly frozen in liquid nitrogen or fixed in 10% formalin solution.

### 2.5 Histological analysis

Tissue sections were fixed in formalin 10% for 24–72 h. After processing and paraffin embedding, staining was done with either Hematoxylin & Eosin (HE) or Masson’s Trichrome for assessing inflammation and collagen deposition, respectively.^59^^,^^60^ Quantifying the tissue staining presented histological grading of inflammation and fibrosis scores.^61^^,^^62^ The slides were seen under light microscope (magnification ×400).

### 2.6 Preparation tissue homogenates

Adhesion bands-linked cecum was collected for making tissue homogenate. The tissue sample (100 mg tissue sample for each animal) was weighted and homogenized with PBS as the homogenization medium. The supernatant of the tissue homogenate was used for the assay of stress oxidative markers.^63^

### 2.7 Measurement of stress oxidative markers

The oxidative stress was evaluated in each subject by measuring the concentration levels of malondiladehyde (MDA) and thiol, as well as catalase activity in the tissue homogenates. All procedures were conducted according to the kit protocol.^64^, ^65^, ^66^

### 2.8 Statistical analysis

Quantitative variables were described as mean ± SEM. One-way ANOVA test was used for comparison between different groups. The collected data were imported into GraphPad Prism for statistical analysis. The statistical significance was set to 5%.

## 3 Results

### 3.1 Saffron and crocin significantly reduced adhesion scores

**Fig. 1:**
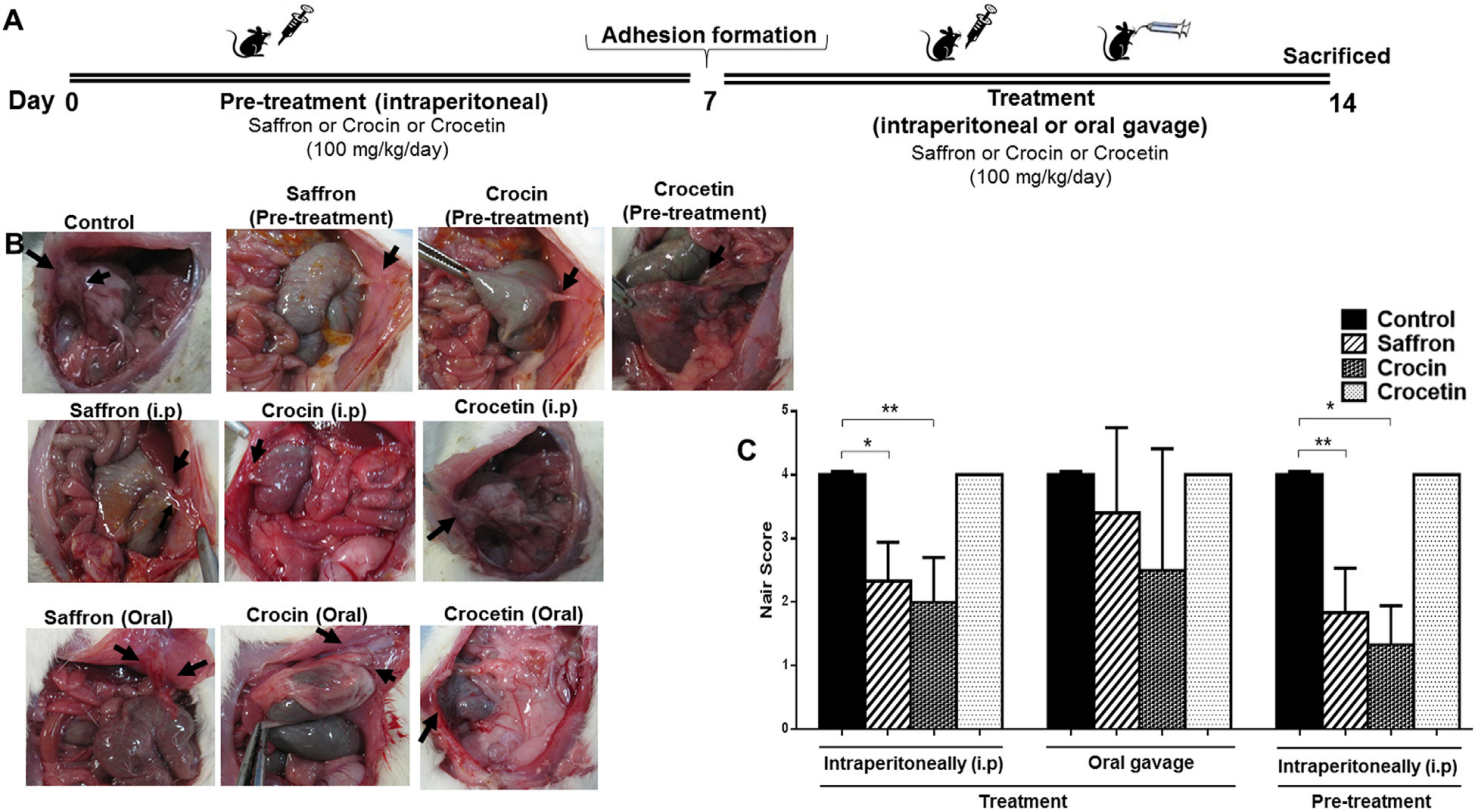
**Saffron and Crocin significantly attenuate adhesion band formation in rat model**. (A) Schematic representation of the experimental protocol. (B) Macroscopic illustrations of different groups of treatment (Identified adhesion bands are shown with arrows). (C) Compared to control group, intraperitoneal treatment or pre-treatment with Saffron (100 mg/kg/day) or Crocin (100 mg/kg/day) significantly reduced the frequency of adhesion bands in rat. Treated mice showed no response to intraperitoneal injection of Crocetin (100 mg/kg/day). The sample size For all groups was n = 6mice/group. ∗p < 0.05; ∗∗p < 0.01; ∗∗∗p < 0.001.

We investigated the therapeutic effect of hydro-alcoholic extracted of saffron, crocin, and crocetin in different administration in adhesion rat model. No animal death occurred during the experiment, and the body weight did not significantly differ after second surgery. There was no infection or bleeding in post-operative time. Schematic representation of the study protocol is shown in Fig. 1A. According to clinical observations, pre-treatment with crocin is the treatment of choice, offering more recovery and reducing the adhesion formation probability (Fig. 1B). We quantitatively analyzed the incidence of adhesion band in different groups, using Nair^54^ scoring scheme (Fig. 1C). Compared to the rat control group, pre-treatment or treatment (i.p) with saffron or crocin significantly improved the frequency of post-operative intra-abdominal adhesion bands (P value < 0.05). Moreover, decrease in adhesion bands frequency (Nair grade) was not significant in oral administration groups. No reduction of adhesion bands were found in the case of crocetin treatment (Fig. 1B and C), compared to control cases. We did not continue experiments on crocetin due to adverse results.

### 3.2 Saffron extract and crocin decreased inflammation in post-surgical adhesion bands

**Fig. 2:**
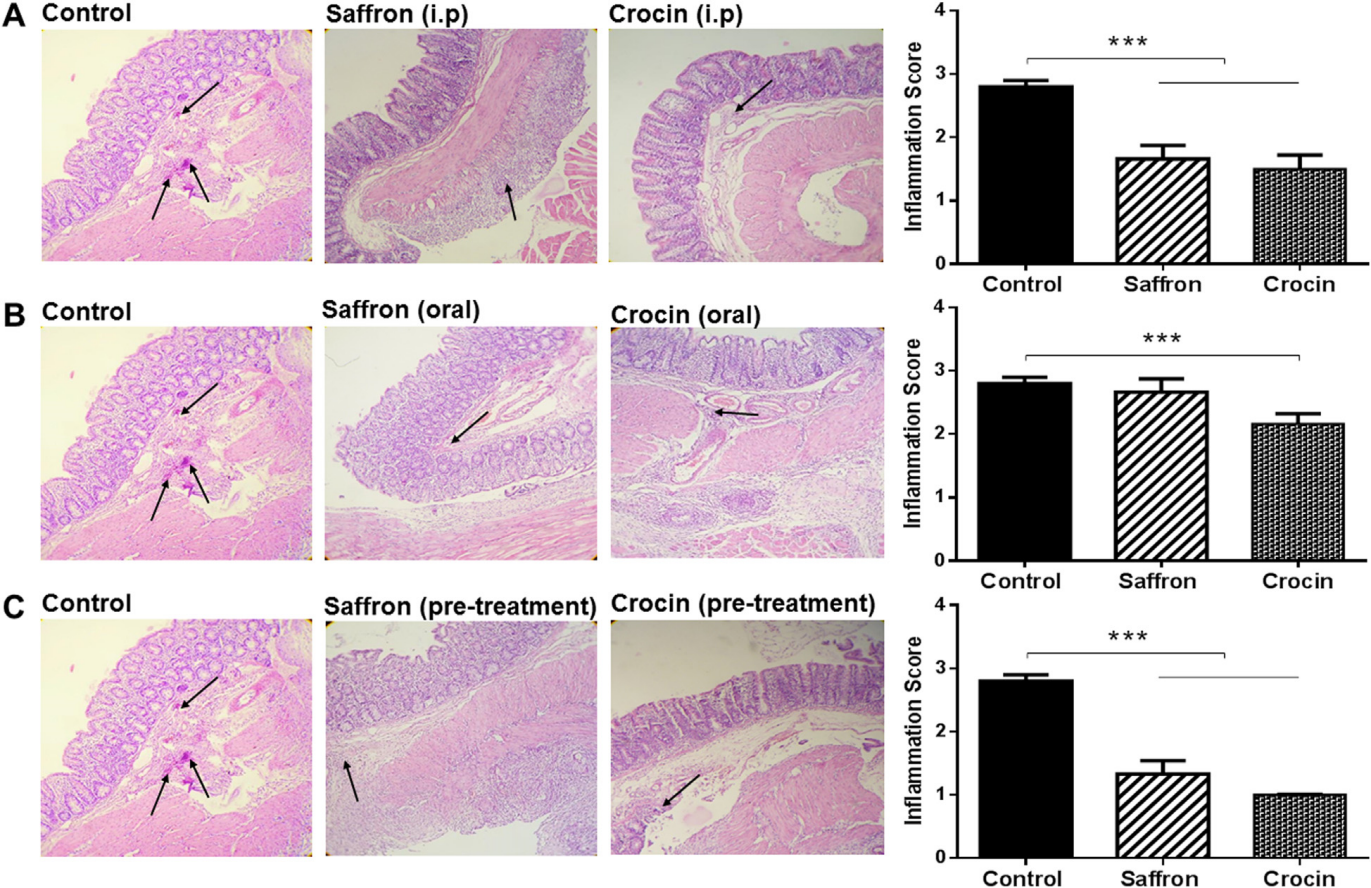
**The inhibitory effects of Saffron and Crocin on inflammation in adhesive tissues**. (A, C) Intraperitoneal injection of Crocin (100 mg/kg/day) or Saffron (100 mg/kg/day) reduced inflammatory cell infiltration (black arrows) in (A) treatment and (C) pre-treatment groups. (B) Despite crocin, oral administration of Saffron showed no significant protective responses in adhesion rat model. ∗∗∗p < 0.001.

Since inflammatory responses are increased during adhesion bands formation,^67^ we evaluated the inhibitory effects of saffron and its pharmacological active components on inflammation in adhesion bands tissues, using Hematoxylin & eosin (HE) staining. Histological analysis and quantitative evaluation showed a significant reduction of inflammation in treatment as well as pre-treatment groups (Fig. 2A–C). As expected, oral treatment with hydro-alcoholic extract of Saffron showed no significant differences, compared to control tissues (Fig. 2C). The detailed of inflammation scoring system is shown in Table 2.

**Table 2:** Infiltration of inflammatory cells and fibrosis scoring according to Swolin.

**Grade**	**Inflammatory cell infiltrate**	**Fibrosis**
**0**	Absent or normal in number	None
**1**	Slight increase	Slight
**2**	Moderate infiltration	Moderate
**3**	Dense	Dense

### 3.3 Saffron and crocin decreased inflammatory responses via reducing oxidative stress markers in post-surgical adhesion band

**Fig. 3:**
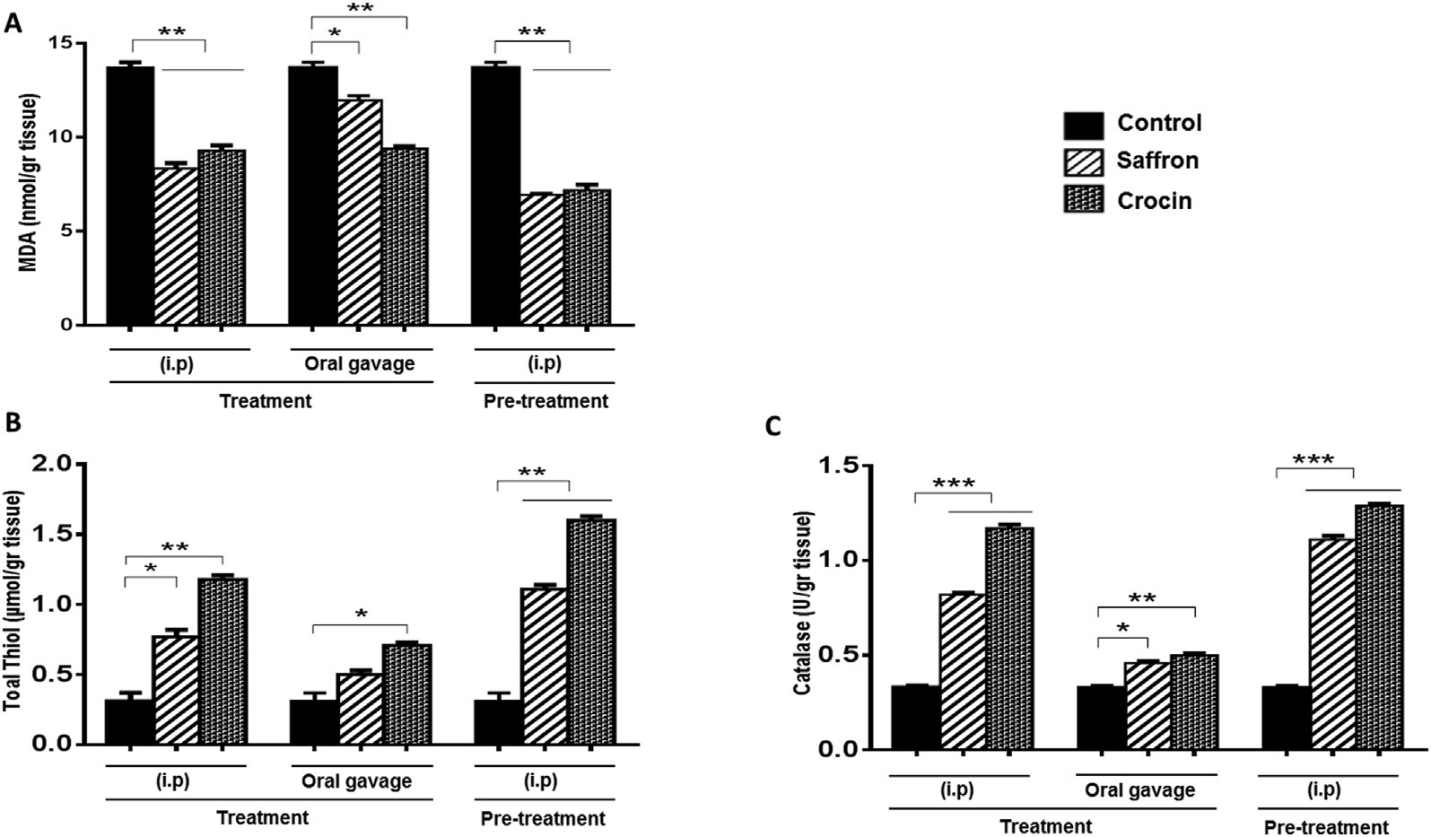
**Oxidative stress is attenuated following Saffron- or Crocin-treatment in adhesive tissues**. (A–C) The tissue concentration of (A) MDA, (B) total Thiol, and (C) the activity of Catalase were compared between different groups. ∗p < 0.05; ∗∗p < 0.01; ∗∗∗p < 0.001.

To further determine the anti-inflammatory mechanisms of Crocin and Saffron in adhesion bands, we measured concentration of oxidant marker, Malon deladehyde (MDA), as well as antioxidants agents including total Thiol concentration and Catalase activity in adhesive tissue homogenates. Our findings showed significant reduction of MDA concentration in all treatment group, compared to control group (Fig. 3A). Consistently, the level of total Thiol (Fig. 3B) and catalase activity (Fig. 3C) were higher in treated groups than control tissues. These results supported the hypothesis that saffron and crocin elicited their protective functions at least partially by attenuating oxidative stress reactions in post-operative adhesion rat model.

### 3.4 Inhibitory effects of saffron and crocin on tissue fibrosis in adhesion rat model

**Fig. 4:**
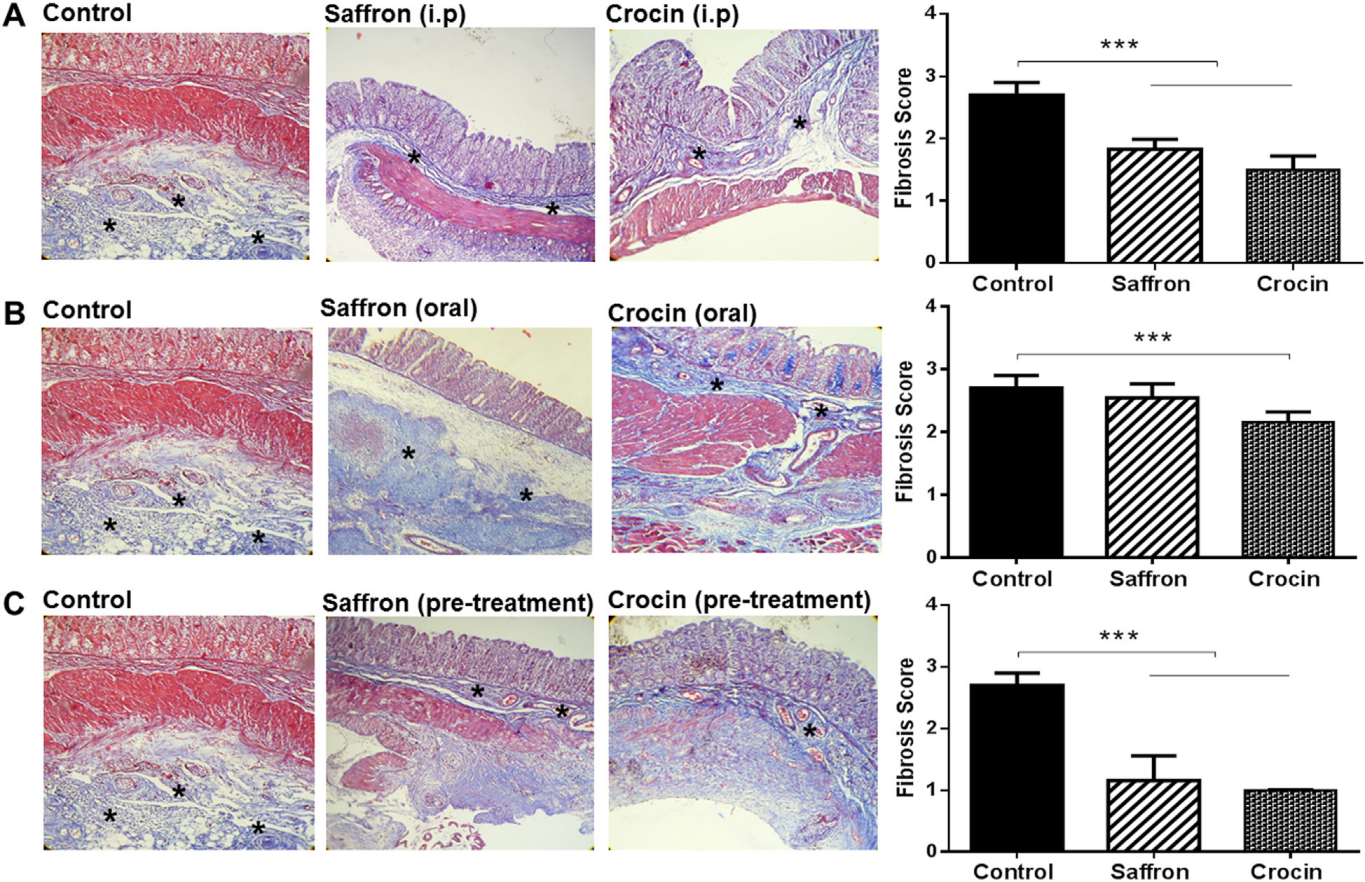
**Saffron and Crocin suppressed fibrosis and Collagenesis in adhesive tissues**. (A, C) Masson’s trichrome staining showed a significant reduction of deposition of Collagen (asterix) in Crocin- or Saffron-treated rat in different groups (B) The decrease in collagen thickening was not significant in the case of oral administration of Saffron, compared to control. ∗∗∗p < 0.001.

Next, we investigated the regulatory effects of saffron and crocin on fibrosis as key factors in the pathogenesis of adhesion band formation. Masson’s trichrome staining accompanied with quantitative analysis demonstrated that crocin or hydro-alcoholic extracted of Saffron could significantly decrease the areas of fibrosis and collagen deposition in adhesive tissues (Fig. 4A–C). The therapeutic effect was more potent in crocin administration group. The fibroblast activity quantified according to the scoring system presented in Table 2. Similar to previous results, oral treatment of Saffron elicited no protective effect in adhesion tissues (Fig. 4B) (P value < 0.05).

## 4 Discussion

The present study investigated the protective effects of hydro-alcoholic extract of saffron, crocin and crocetin on intra-abdominal adhesion models, using different administration methods. Macroscopic results demonstrated a significant reduction of adhesion bands frequency in intraperitoneal administration of saffron or crocin as well as the pre-treatment group. However, neither crocetin, nor oral administration of saffron could prevent post-surgical adhesion band formation in rat model. We also showed that decrease in the oxidative stress responses as well as attenuating fibrosis and collagen depositions are some of the mechanisms by which saffron and crocin (i.p) exert their protective responses in adhesion model, supporting the therapeutic potential of these low-toxic compounds against post-surgical adhesion band formation.

Damage to peritoneum leading to the deposition of peritoneal fibrin and local inflammation are the key steps of adhesiogenesis.^9^ Studies have shown that using fibrinolytic compounds prevent the progress of adhesion formation via stimulation of the intraperitoneal fibrinolytic system.^68^, ^69^, ^70^ Moreover, it has been found that adhesion formation is accompanied by lower tissue oxygenation and free oxygen radical generation.^71^^,^^72^ Ezberci et al.^73^ showed that decrease in oxidant agent including MDA whereas increase in catalase activity attenuated adhesion score in Bacterial peritonitis rat model. Consistent with these results, our previous finding revealed that decrease in oxidative stress markers, as well as collagen deposition and infiltration of inflammatory cells to injured site attenuated the severity of adhesions in animal model.^14^ Also, we showed that human APC significantly reduced the formation of adhesion bands which is correlated with lower concentration of pro-inflammatory cytokines and higher tissues plasminogen activator (tPA) *in vivo*.^15^

Furthermore, there are several studies supporting the anti-oxidative, anti-inflammatory, and anti-fibrotic properties of saffron and crocin in different models. For instance, Hemshekhar et al.^74^ showed that crocin enhanced anti-oxidant status by increasing catalase, and superoxidase dismutase (SOD) activities as well as attenuating serum level of inflammatory factors in arteritis rat model. Moreover, Samarghandian et al.^75^ reported that crocin improved aged rat kidney functions by reducing oxidative stress and inflammatory responses in rat. Consistently, Hashemi et al. indicated the effects of saffron carotenoids, crocin and crocetin on oxidative stress in breast tumor. They showed that crocin and crocetin increase the catalase^76^ and superoxide dismutase^77^ activities in BALB/c mice, after 28 days of treatment. Since crocin treatment has been shown antioxidant activities under various conditions, Nasimian et al. showed that crocin elevated the apoptotic death of human breast cancer cell lines, partially via ROS-activated FOXO3a axis.^78^ Moreover, clinical trial studies evaluated the effect of saffron aqueous extract, crocin^79^ and crocetin^80^ in coronary artery disease (CAD). It has been shown that crocin and crocetin treatment resulted in a significant reduction in lectin-like oxidized LDL receptor 1 (LOX1), nuclear factor kappa-B (NF-κB) and Serum ox-LDL. Also, the levels of monocyte chemoattractant protein 1 (MCP-1) was reduced in all treatment groups in CAD patients.^79^^,^^80^ Similarly, we recently showed that crocin significantly inhibited oxidative stress and histopathological scores, representing a reduction of inflammation and fibrosis in dextran sodium sulfate (DSS)-induced colitis model.^45^ Another study showed that 100 mg/kg of saffron reduced MDA, Myeloperoxidase, and tumor-necrosis factor-alpha (TNF-α) in pulmonary fibrosis.^81^

Consistent with the fibrinolysis effect of crocin in post-surgical adhesion model, it has been shown that saffron could significantly prevent the thickness of alveolar septa and collagen deposition in bleomycin-induced pulmonary fibrosis.^81^ In line with these results, Mehrabani et al. demonstrated the protective effects of crocin against fibrosis and hydroxyproline content of lungs.^82^ Chhimwal et al. showed that crocin decreased the hepatic fibrosis via expression of peroxisome proliferator-activated receptor γ (PPAR-γ), modulating the inflammatory and fibrogenic pathways.^83^ These results are consistent with another study showing the anti-fibrotic and anti-inflammatory properties of crocin in thioacetamide (TAA)-induced liver fibrosis.^47^

In conclusion, this study suggested that saffron and crocin have potential therapeutic value in preventing intra-abdominal adhesion band formation. The mechanism underlying abdominal adhesions has not yet been completely understood. Further animal and clinical studies are required to clarify this issue and to assess the exact mechanism of action for Saffron and its pharmacologically active component, crocin, for preventing adhesion bands formation.

## Data availability statement

Research Data are not shared.

## Declaration of competing interest

The authors have no conflicts of interest.
